# Sex impact on daily activity and physiological, metabolic and hormonal responses to different photoperiod regimens in diet-induced obese fischer 344 rats

**DOI:** 10.1007/s13105-025-01075-w

**Published:** 2025-06-10

**Authors:** Saioa Gómez-Roncal, Aina Gironès-Garreta, Manuel Suárez, Begoña Muguerza, Gerard Aragonès, Miquel Mulero, Anna Arola-Arnal

**Affiliations:** 1 Departament de Bioquímica i Biotecnologia, Nutrigenomics Research Group, Universitat Rovira i Virgili, C/ Marcel·lí Domingo 1, Tarragona, 43007 Spain; 2 Nutrigenomics Research Group, Institut d’Investigació Sanitària Pere Virgili, C/ Marcel·lí Domingo 1, Tarragona, 43007 Spain; 3 Center of Environmental, Food and Toxicological Technology (TecnATox), University Rovira i Virgili, C/ Marcel·lí Domingo 1, Tarragona, 43007 Spain; 4 CIBERobn Physiopathology of Obesity and Nutrition, Institute of Health Carlos III, Madrid, Spain

**Keywords:** Cafeteria diet, Female, Male, Metabolic syndrome, Circadian rhythms, Season

## Abstract

Seasonal variations in terms of light length per day significantly influence physiological, behavioral, and metabolic processes in mammals, which are crucial for adaptation to environmental changes. This study examines the sex-dependent responses to different photoperiods on diet-induced obese Fischer 344 (F344) rats, focusing on body weight, daily activity, metabolism, and hormone levels. Male and female F344 rats were fed a cafeteria diet for 11 weeks and exposed to short (L6, 6 h of light per day) and long (L18, 18 h of light per day) photoperiods to mimic winter and summer conditions during the last 8 weeks.

Results indicated that male rats exhibited greater body weight gain compared to females under both photoperiods, with a more pronounced increase in the long photoperiod correlating with testosterone levels. In addition, females displayed different activity distributions than male rats in L6 conditions. Male and female rats had a similar lipid profile in both photoperiods, but females presented a better lipid profile and insulin sensitivity, correlating with increased levels of melatonin. There is a sex-dependent effect on glucose levels only in short photoperiod with reduced levels in female rats. In conclusion, there is an interaction between seasonal rhythms and sex, which results in varying susceptibilities to diet-induced obesity. This research underscores the importance of considering sex differences in obesity studies and metabolic responses to different light conditions.

## Introduction

In mammals, seasonal changes in metabolism, reproduction and behavior are processes that require long-term changes, thus animals can anticipate and adapt in advance to those changes in their environment [1]. These adaptive mechanisms are crucial for survival and reproductive success in environments with predictable annual variations in climate, food availability, and other resources [2]. Those seasonal changes, also known as seasonal rhythms, are 12-month duration cyclical biological processes that regulate various physiological functions [1, 3]. These rhythms are controlled by complex interactions between environmental cues, mainly light, endogenous circannual clocks, and neuroendocrine pathways [4].

The seasonal regulation of body weight and metabolism involves neuroendocrine mechanisms, particularly the hypothalamus-pituitary-thyroid (HTP) axis [5]. In fact, hormones such as melatonin or corticosterone are strongly regulated by changes in day length [6]. Melatonin, released in the dark phase, is considered an internal neurochemical representation of photoperiods (daylight lengths) and the main reason for the photoperiodic response [4]. Melatonin influences photoperiodic processes by its action on pars tuberalis of the pituitary gland, which regulates thyroid-stimulating hormone (TSH). This hormone controls the activation of thyroid hormone in hypothalamus by controlling deiodinase enzymes type 2 and 3 (Dio2, Dio3), that are able to activate or inactivate thyroid hormone, respectively [4, 5, 7].

Photoperiodic changes significantly influence the physiological responses, behavior and gene expression in rodents, particularly regarding weight and metabolic regulation [7, 8]. It is well described that in rats, long photoperiods (more than 12 h of light per day) exert a stimulatory response in terms of body weight gain and food intake, while in short photoperiods (less than 10 h of light per day) an inhibitory effect can be seen, also in terms of hypothalamic gene expression [7]. Interestingly, studies in humans have shown that seasonal variations in day length can affect mood, appetite, and energy expenditure, which may contribute to seasonal patterns in weight gain and metabolic health [9].

Obesity is a risk factor for many metabolic diseases, such as hypertension, type 2 diabetes, cardiovascular diseases, or dyslipidemia, which can lead to the development of metabolic syndrome (MetS) [10–12]. Obesity is high prevalent nowadays and it is considered a seasonal rhythm disruptor by altering metabolic processes such as lipid metabolism or hormone production [13]. Photoperiod-induced metabolic changes show significant variations in obesity models. In rodents, short photoperiods typically lead to decreased food intake and increased energy expenditure, reflecting a catabolic state. In contrast, long photoperiods are linked to an anabolic profile, characterized by higher food intake, lower energy expenditure, and increased adiposity [14]. These photoperiodic effects are often exaggerated or disrupted in obese models, further complicating metabolic adaptations. The altered seasonal signaling observed in obese animals is associated with changes in hormone profiles, including melatonin and leptin, as well as shifts in inflammatory markers [14, 15].

Several studies have shown that rats fed with high fat diets gain weight and induces a status of dyslipidemia and creates insulin resistance by altering glucose metabolism [13, 16]. It has been reported that changes induced by a high-fat diet can affect corticosterone levels [17], energy expenditure, and expression levels of orexigenic genes [13]. In this sense, cafeteria (CAF) diet has been proven to be a good model to study all those changes in rodents, as it can develop MetS in rats very similar to human, allowing the results to be properly extrapolated [18].

Sex differences in obesity among rats have been extensively studied, revealing notable distinctions in how each sex responds to obesogenic diets and develops metabolic disturbances. Specifically, it has been shown that the food intake and the body weight gain is different between sexes, accumulating males more visceral fat when fed with high-fat diets than females, despite the adiposity index is lower [19]. Other studies point out the sex-differences in metabolic disturbances, such as females having better insulin sensitivity, suggesting a hormone protective mechanism and highlighting the role of estrogen [20]. Recent studies have demonstrated that male and female rats exhibit distinct patterns of gene expression in adipose tissue and liver in response to high-fat diets, which may contribute to the observed sexual dimorphism in obesity development [21]. Furthermore, sex-specific differences have been observed in the hypothalamic regulation of energy balance and in the response to various obesity interventions [22]. For instance, female rats have shown greater resistance to diet-induced obesity and better maintenance of glucose homeostasis compared to males [23]. Additionally, the impact of obesity on cardiovascular function and inflammation has been found to be sex-dependent, with males often showing more severe consequences [24]. These differences underscore the critical importance of studying the effect of sexes in obesity research to fully understand the complex interplay between sex, hormones and metabolism. Several studies have investigated the changes occurring in healthy animals under different photoperiods, as well as in models of obesity. However, to our knowledge, the interaction between sex and photoperiod has not been investigated.

Taking all together, we hypothesize that the susceptibility to diet-induced obesity is modulated by an interaction between photoperiod and sex, with males and females exhibiting different responses to varying daylight lengths that influence metabolic and physiological factors. Hence, this study aims to evaluate the sex-dependent effects upon exposure to long and short photoperiods in diet-induced obese photoperiod-sensitive Fischer 344 (F344) rats, focusing on changes in body weight, daily activity, metabolism and hormone levels. For this purpose, male and female F344 rats were chronically fed a CAF diet and exposed to a short photoperiod (L6, 6 h of light per day) and a long photoperiod (L18, 18 h of light per day) to mimic the day length of winter and summer seasons, respectively.

## Materials and methods

### Experimental procedure in rats

A total of 40 male and female (*n* = 20) F344 rats (12-week-old) were purchased from Charles Rivers Laboratories (Barcelona, Spain) and housed by pairs at 22ºC and 50% humidity under standard conditions of light and darkness (L12, 12 h light/day), on reverse light cycle (lights off at 8 a.m.). The sample size of each group has been calculated to detect differences in mean fasting blood glucose levels. A power analysis was performed based on an alpha significance level of 0.05 and a beta of 0.2 to achieve 80% power to detect a 20–30 mg/dL difference in mean fasting blood glucose levels [25]. Thus, a sample size of 10 animals per group will be sufficient to detect clinical differences.

**Fig. 1:**
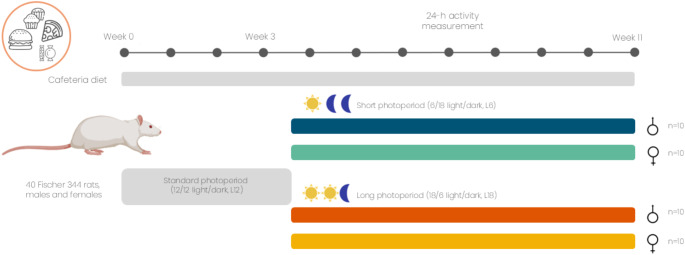
Animal experimental design. 20 male and 20 female F344 rats were fed with CAF diet for 11 weeks and exposed to a short photoperiod of 6/18 h light/darkness (L6) and long photoperiod of 18/6 h light/darkness (L18) for the last 8 weeks (*n* = 10). Activity was measured at week 7

The animals were fed daily with CAF diet and tap water ad libitum during the experiment. The CAF diet calorie breakdown was 14% of proteins; 35% of fat and 51% of carbohydrates and consisted of bacon, *ensaïmada* (pastry), biscuits with cheese and pâté, carrots, standard chow, and milk with 22% sucrose (w/v). Diet was newly introduced to the animals one hour after the light turned off. After 3 weeks of diet adaptation, 10 animals per sex were exposed to L6 photoperiod and 10 animals per sex to L18 photoperiod for 8 weeks (Fig. 1). No standard photoperiod L12 was used because it is well reported that photoperiods with more than 10 h of light per day exert a stimulatory response in terms of body weight gain and food intake [7]. Animal weight and food intake were weekly recorded during the whole experiment. For food intake recording, the weight of each dietary component was measured before and after a period of 24 h. Food intake was estimated by assuming equal consumption among the two rats within each cage, and the total intake of Kcal was calculated based in the nutritional information provided by the correspondent manufacturer. Animal activity was measure during a 24 h period to analyze their daily circadian cycle of activity, including the active and phase rest, at week 7 of the experiment, once rats were totally adapted to their photoperiods. The last day of the experiment, food was removed when the lights turned off, and 3 h later, at 11 a.m., animals were sacrificed by decapitation. Blood was collected immediately in heparinized tubes, and centrifuge at 1200x g for 15 min to collect the plasma, and different organs and tissues were dissected and weighted.

Adiposity index was calculated by dividing the sum of the weights of white adipose tissues by the final body weight as a percentage. Fat mass was calculated as the sum of white adipose tissues (epidydimal or periovarian, mesenteric and inguinal), perirenal and subcutaneous adipose tissues. Lean mass tissue was calculated as the difference between body weight and adipose tissues. Energy efficiency coefficient (EEC) was calculated to consider the caloric intake influences on the weight gain, where EEC = total body weight gain / total energy intake, as described by Caroline de Oliveria Melo et al. [26].

Animal experiments were conducted in accordance with the European Communities Council Directive (86/609/EEC) and approved by the Animal Ethics Review Committee for Animal Experimentation of the Universitat Rovira i Virgili and Generalitat de Catalunya (permission number 11610, approved in 2022).

### Animal activity

Animal activity was measured using an Oxylet Pro System (Panlab, Barcelona, Spain) and the Metabolism 2.1.02 (Panlab, Cornellà, Spain) software program. The activity of the rats was recorded with sensors that detected when rat moves in the cage.

### Biochemical parameters in plasma

Glucose level was measured with an enzymatic colorimetric assay (QCA, Amposta, Tarragona, Spain); total non-esterified fatty acids (NEFA) were analyzed using FujiFilm commercial kit (WAKO, Neuss, Germany); total cholesterol (TC), high-density lipoprotein (HDL-C), low-density lipoprotein (LDL-C), and triglycerides (TAG) were analyzed in an automatic analyzer Cobas Mira Plus Autoanalyzer (Roche Diagnostics, Spain) using commercial kits (Spinreact SA, Spain).

HDL-C/LDL-C ratio, TC/HDL-C ratio were calculated in order to assess the risk for the development of cardiovascular disease. To evaluate the impact of CAF diet on cardiovascular risk, atherogenic index (AI) was estimated as AI = log [TAG/HDL-C], as described by Caroline de Oliveira Melo et al. [26].

Leptin and insulin levels were quantified using enzyme-linked immunosorbent assays (ELISA) kits for rat and mouse (EMD Millipore Corporation, St. Louis, Missouri). The homeostatic model assessment for insulin resistance (HOMA-IR) index was calculated as fasting insulin levels (IU/mL) x fasting glucose levels (mg/dL)/405. The visceral hypertriglyceridemic phenotype (VHP) was calculated to evaluate another marker of insulin resistance, which is associated with nutritional imbalances [26]. VHP was calculated as: VHP = [ln (TAG x glucose)/2] x visceral fat.

### Circulating hormones

Circulating hormones melatonin, corticosterone, testosterone, triiodothyronine (T3), and tetraiodothyronine (T4) were analyzed using liquid chromatography with triple quadrupole tandem mass spectrometry (LC-QqQ-Ms/Ms). For this, 50 µL of plasma was extracted with 250 µL of ethanol containing the internal standard (2 ng/ml). After centrifugation during 5 min 4 ◦C and 252× g, the supernatant was transferred to a new tube and mixed with 700 µL of 0.1% formic acid in water and the sample was loaded to a solid-phase extraction (SPE) system conditioned with methanol and 0.1% formic acid in water. The cartridge was washed with 0.1% formic acid in water and, after dry under high vacuum, the compounds were eluted with 500 µL of methanol and samples were evaporated in a Speed Vacuum at 45 ◦C and reconstituted with 50 µL of water: methanol (60:40, v/v). After the extraction, samples were analyzed using a Zorbax Eclipse C18 column (150 × 2.1 mm) from Agilent Technologies.

### Statistical analysis

Results are presented as median (quartile 1 (Q1)– quartile 3 (Q3)) or box plots with whiskers showing minimum to maximum values, median and interquartile range. Statistical analysis was performed using GraphPad Prism software (V9; Dotmatics). Sample size lower than 15 was taken as non-parametric samples. In comparisons between > 2 groups, two-way analysis of variance (ANOVA) followed by Tukey HSD multiple comparisons test were performed. Two-sided *p*-values of < 0.05 were considered statistically significant, and *p*-values between 0,1 and 0,05 were considered as a tendency. Correlation analysis between different hormone levels and other parameters were also carried out using GraphPad Prism software (V9, Dotmatics).

## Results

### Photoperiods regulates fat mass, energy efficiency and activity in a sex-dependent manner

**Fig. 2:**
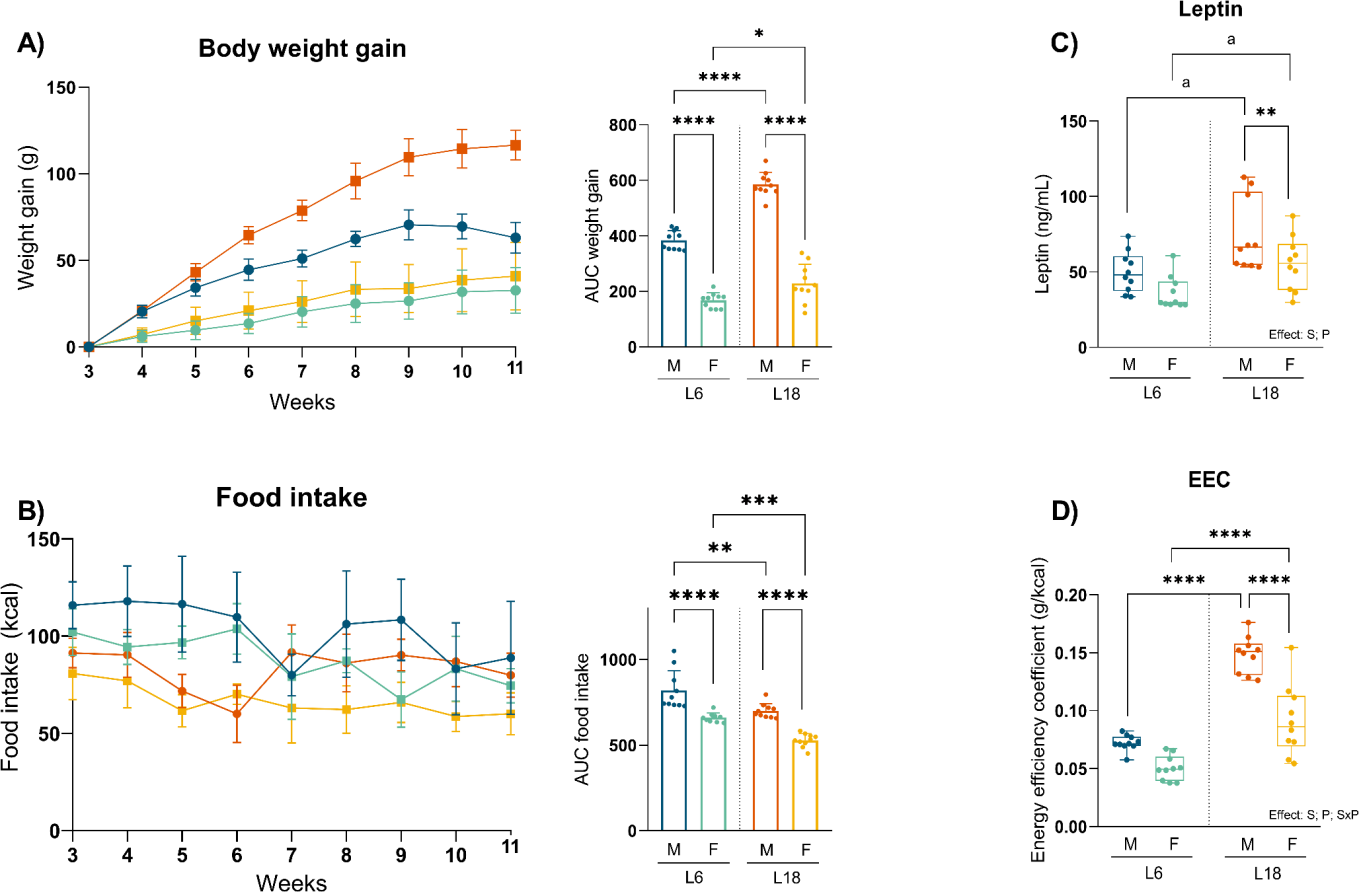
Effect of photoperiod on body weight gain (**A**), daily food intake (**B**), leptin levels (**C**) and on energy efficiency coefficient (**D**) in males and females F344 rats fed with CAF diet for 11 weeks and exposed for the last 8 weeks to two different photoperiods: short photoperiod of 6/18 h light/darkness (L6) and long photoperiod of 18/6 h light/darkness (L18). Data are expressed as mean ± SEM (*n* = 6–10) in AUC graphs, and as minimum to maximum values, median and interquartile range (*n* = 10) in leptin and energy efficiency coefficient. S, sex effect; P, photoperiod effect; SxP, interaction between sex and photoperiod using 2-way ANOVA and Tukey as post-hoc comparisons; * Indicates significant differences by groups (* *p* < 0,05, ** *p* < 0,01, *** *p* < 0,001, **** *p* < 0,0001); a Indicates trend (*p* = 0.1–0.051) using 2-way ANOVA and Tukey as post-hoc test. *AUC*, area under the curve; *M*, males; *F*, females

In both photoperiod exposure conditions, male rats had higher body weight gain than female rats (Fig. 2A). Also, male rats had higher liver and lean/fat mass ratio than females. However, adiposity index was higher in female rats in both photoperiods (Table 1). Otherwise, both males and female rats exposed to a L18 photoperiod had a higher body weight gain and EEC than their counterparts exposed to a L6 photoperiod. Even though, in males the increase in body weight gain between photoperiods (52.35%) was most important than in females (36.79%). Although there is not a photoperiod effect, female rats in L6 had increased fat mass and adiposity index and decreased lean mass than males. There was a photoperiod effect on leptin levels, which were higher in males and females exposed to L18 photoperiod. In addition, leptin levels in L18 were higher in male than in female’s rats (Fig. 2C). Male rats had higher food intake than female rats in both photoperiod exposure conditions (Fig. 2B). Food intake was lower in male and female rats exposed to L18 photoperiod, despite no differences could be observed throughout each week. In addition, a significant interaction between sex and photoperiod was observed in EEC, indicating that the photoperiod’s effect on caloric intake influences weight gain in a sex-dependent manner.

**Table 1:** Sex differences between photoperiods in physiological parameters

	L6 photoperiod		L18 photoperiod		
	**Males**	**Females**	**Males**	**Females**	**Effect**
Final body weight (g)	380.5 (373.3–397.5)	230.5 (224.8–240.0) ^***^	446.0 (431.8–458.3) ^&&&^	248.5 (240.5–275.5) ^***^	S, P, SxP
Liver (%)	2.792 (2.714–2.981)	2.291 (2.227–2.363) ^***^	3.101 (2.987–3.182) ^&&^	2.347 (2.310–2.429) ^***^	S, P, sxp
Brain (%)	0.493 (0.489–0.528)	0.758 (0.723–0.787) ^***^	0.444 (0.439–0.452) ^&&&^	0.725 (0.683–0.782) ^***^	S, P
Cecum (%)	1.110 (0.945–1.316)	1.142 (1.006–1.371)	0.899 (0.836–0.984) ^b^	0.918 (0.827–1.051) ^&^	*P*
Gastrocnemius and soleus muscles (%)	0.455 (0.434–0.472)	0.474 (0.458–0.489)	0.379 (0.355–0.415) ^&&&^	0.457 (0.439–0.509) ^***^	S, P, SxP
IBAT (%)	0.159 (0.154–0.176)	0.214 (0.204–0.244) ^*^	0.145 (0.121–0.175)	0.145 (0.128–0.170) ^&&&^	S, P, SxP
Perirenal adipose tissues (%)	3.588 (3.512–3.811)	3.549 (3.465–3.749)	3.522 (3.433–3.728)	3.451 (3.270–3.674)	
Subcutaneous adipose tissues (%)	5.589 (4.857–6.079)	5.742 (5.500–6.306)	6.173 (5.608–6.935)	5.235 (4.638–6.302)	SxP
Adiposity index (%)	16.347 (15.655–18.932)	20.780 (19.775–21.917) ^***^	18.792 (17.334–19.978)	21.424 (20.058–22.615) ^a^	S
Fat mass (%)	16.347 (15.644–18.932)	20.780 (19.758–21.917) ^**^	18.792 (16.959–19.978)	21.424 (19.376–22.615)	S
Lean mass (%)	83.512 (80.923–84.172)	79.010 (77.867–80.008) ^***^	81.059 (79.869–82.944)	78.420 (77.215–80.487)	S
Lean/fat mass ratio	5.114 (4.275–5.381)	3.803 (3.553–4.048) ^***^	4.314 (4.004–4.891)	3.663 (3.420–4.170)	S

Data are expressed as median (quartile 1 (Q1)– quartile 3 (Q3)) (*n* = 10). * Indicates significant differences by sex, & indicates significant differences by photoperiod, a and B indicate tendency respectively. The “effect” column represents the statistical results of photoperiod (P), sex (S) or their interaction (SxP). Upper case represents significant differences (*p* < 0,05) and lower case represents tendency (*p* < 0,1). 2-way ANOVA test and Tukey as post-hoc comparisons (* or & *p* < 0.05, ** or && *p* < 0.01, *** or &&& *p* < 0.001) was used. IBAT, interscapular brown adipose tissue

**Fig. 3:**
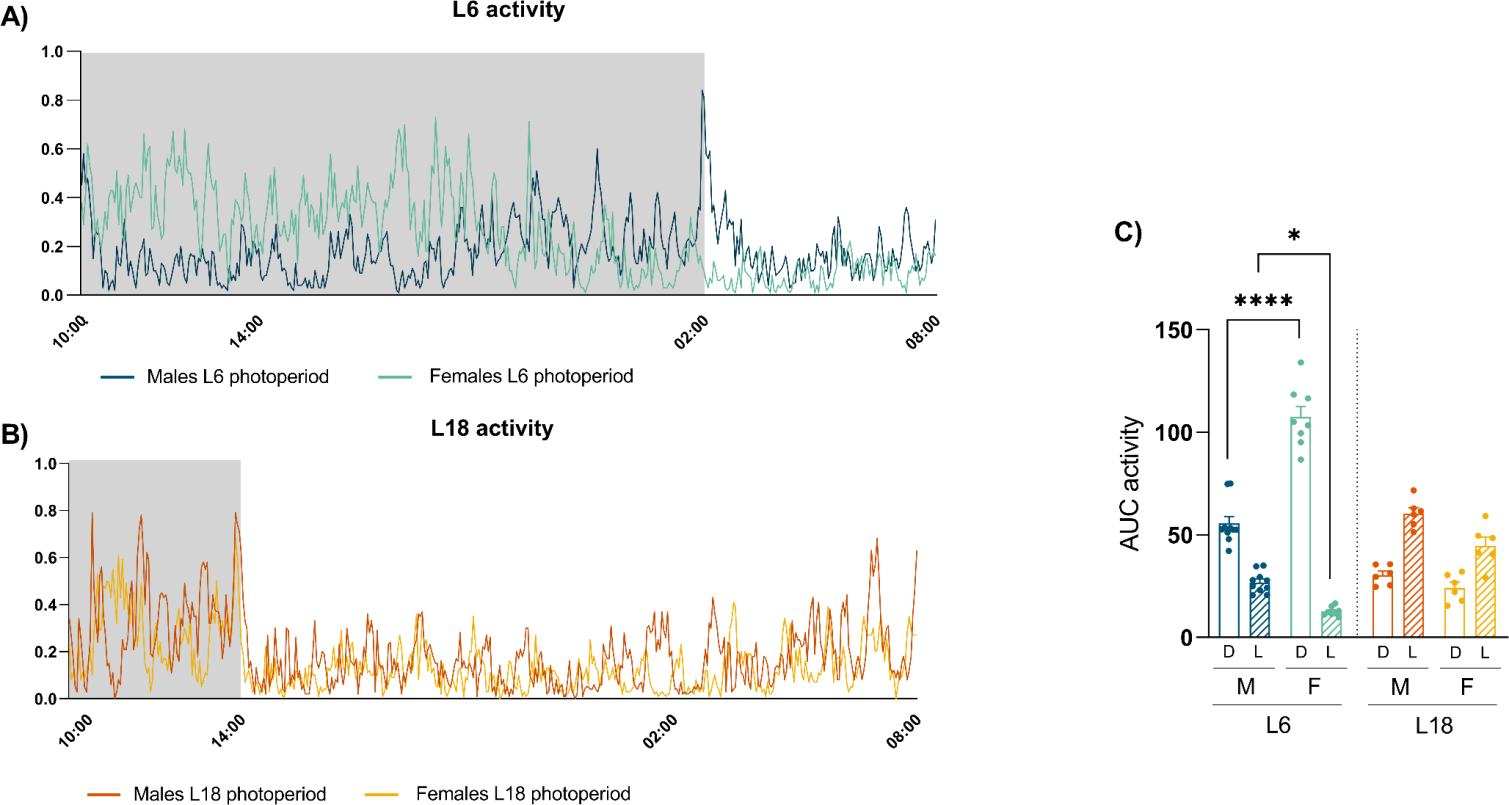
Effect of photoperiod on 24-hours activity in males and females F344 rats fed with CAF diet for 11 weeks and exposed for the last 8 weeks to two different photoperiods: short photoperiod of 6/18 h light/darkness (L6) (**A**), and long photoperiod of 18/6 h light/darkness (L18) (**B**). Area under the curve (*AUC) in* dark (D, active phase) or light (L, rest phase) (**C**). Data are expressed as mean ± SEM (*n* = 6–10). * Indicates significant differences by groups (* *p* < 0,05, **** *p* < 0,0001) using 2-way ANOVA and Tukey as post-hoc test. *M*, males; *F*, females

Regarding the results of activity, males and female rats were more active during the L6 photoperiod as the rats’ active phase last longer. Also, male and female rats had a similar activity during the L18 photoperiod. However, female rats exposed to a L6 photoperiod had higher activity during the active phase and lower activity during the rest phase than male rats (Fig. 3).

### Lipid and glycemic parameters in plasma are modulated by sex and photoperiod

**Fig. 4:**
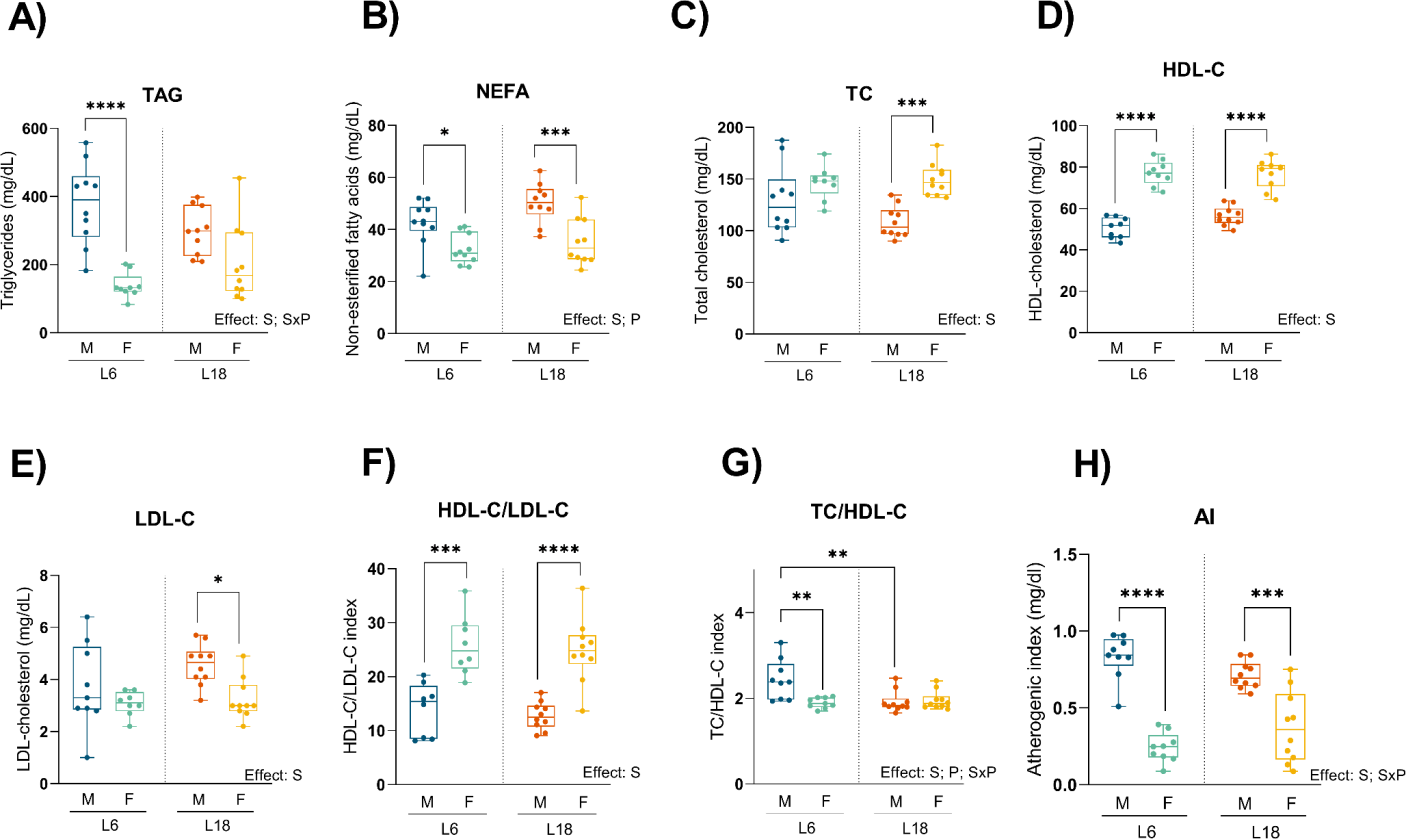
Effect of photoperiod on lipid plasma profile. TAG (**A**), Non-esterified fatty acids (NEFA) (**B**), TC (**C**), high density lipoprotein cholesterol (HDL-C) (**D**), low density lipoprotein cholesterol (LDL-C) (**E**), HDL-C/LDL-C index (**F**), TC/HDL-C index (**G**), and AI (**H**) in males and females F344 rats fed with CAF diet for 11 weeks and exposed for the last 8 weeks to short photoperiod of 6/18 h light/darkness (L6) and long photoperiod of 18/6 h light/darkness (L18). Data are expressed as minimum to maximum values, median and interquartile range (*n* = 10). S, sex effect; P, photoperiod effect; SxP, interaction between sex and photoperiod using 2-way ANOVA and Tukey as post-hoc comparisons; *Indicates significant differences by groups using 2-way ANOVA and Tukey as post-hoc comparisons (* *p* < 0,05, ** *p* < 0,01, *** *p* < 0,001, **** *p* < 0,0001). *M*, males; *F*, females

There was a strong sex-effect on lipid parameters (Fig. 4). In this sense, males had higher levels of NEFA and lower levels of HDL-C than female rats in both photoperiod conditions. Also in both photoperiods, the HDL-C/LDL-C index was lower in males, while AI was higher in females. In NEFA and AI there was a photoperiod effect or sex-photoperiod interaction, respectively. However, female rats only when exposed to L18 photoperiod had increased levels of TC and decreased levels of LDL-C than males exposed to the same photoperiod. On the other hand, only when exposed to L6 conditions, females had lower levels of TAG than male rats in L6. Finally, males exposed to L18 had a lower TC/HDL-C index than when exposed to L6 and this index in males was higher than in females exposed to L6.

**Fig. 5:**
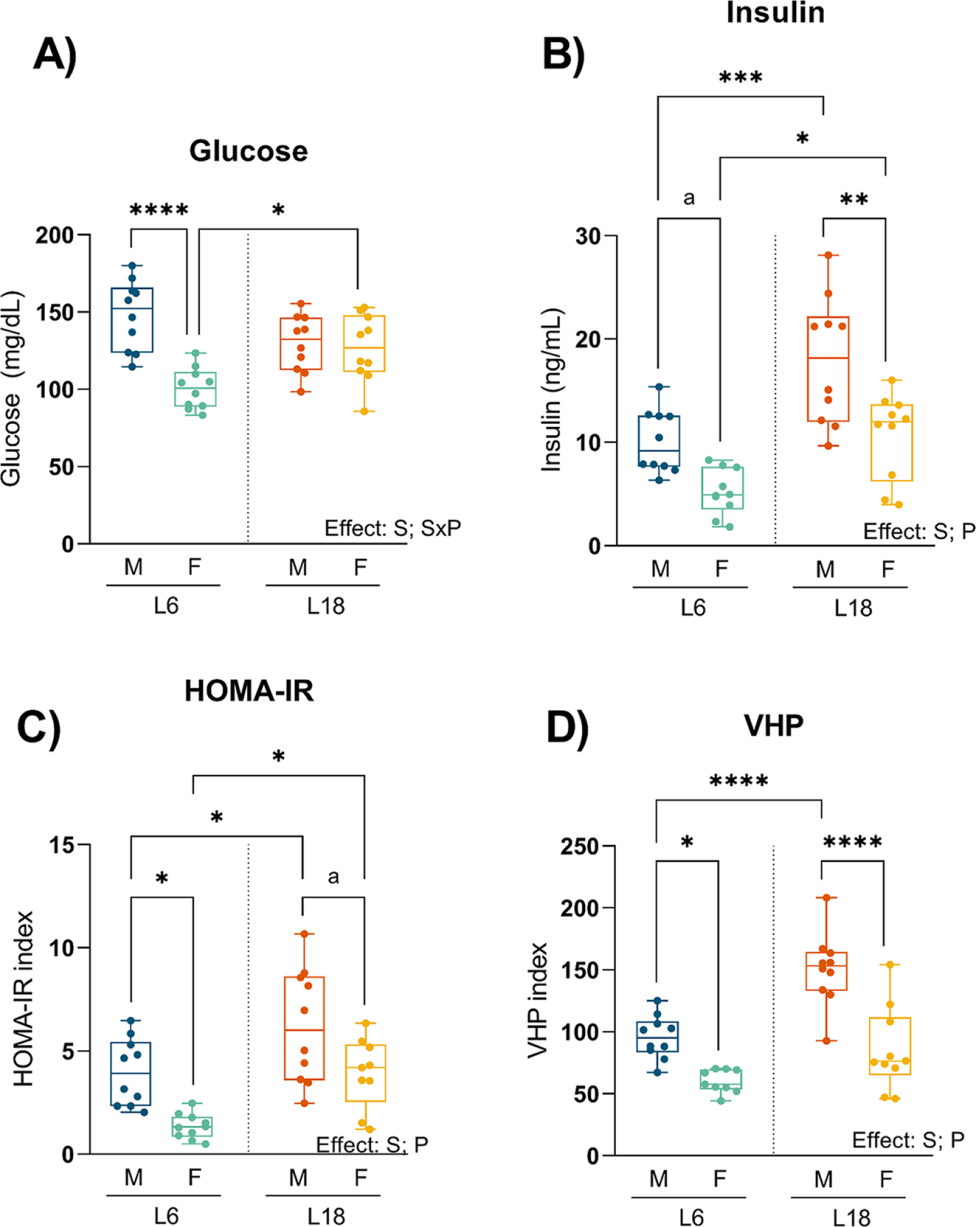
Effect of photoperiod on glycemic parameters in plasma. Glucose in plasma (**A**), insulin (**B**), homeostasis model assessment for insulin resistance (HOMA-IR index) (**C**), and VHP (**D**) in males and females F344 rats fed with CAF diet for 11 weeks and exposed for the last 8 weeks to short photoperiod of 6/18 h light/darkness (L6) and long photoperiod of 18/6 h light/darkness (L18). Data are expressed as minimum to maximum values, median and interquartile range (*n* = 10). S, sex effect; P, photoperiod effect; SxP, interaction between sex and photoperiod using 2-way ANOVA and Tukey as post-hoc comparisons; * Indicates significant differences by groups (* *p* < 0,05, ** *p* < 0,01, *** *p* < 0,001, **** *p* < 0,0001); a Indicates trend (*p* = 0.1–0.051) using 2-way ANOVA and Tukey as post-hoc test. *M*, males; *F*, females

Sex and photoperiod also modulate plasma glycemic parameters (Fig. 5). Male rats exposed to L6 and L18 photoperiods showed increased values of the evaluated glycemic parameters than females exposed to the same photoperiods, except for glucose in L18, with similar levels in both sexes. Interestingly, rats responded differently to photoperiod conditions but similarly between sexes. In this regard, male and female rats had a different response to photoperiod conditions in insulin and HOMAR-IR with increased values in L18. However, glucose levels were similar in males exposed to L6 and L18 conditions, but females had decreased levels in L6. In VHP, males but not females’ rats had higher levels in L18 photoperiod.

### Hormone levels in plasma are sex-dependent in both photoperiods, but only photoperiod-dependent in males

**Fig. 6:**
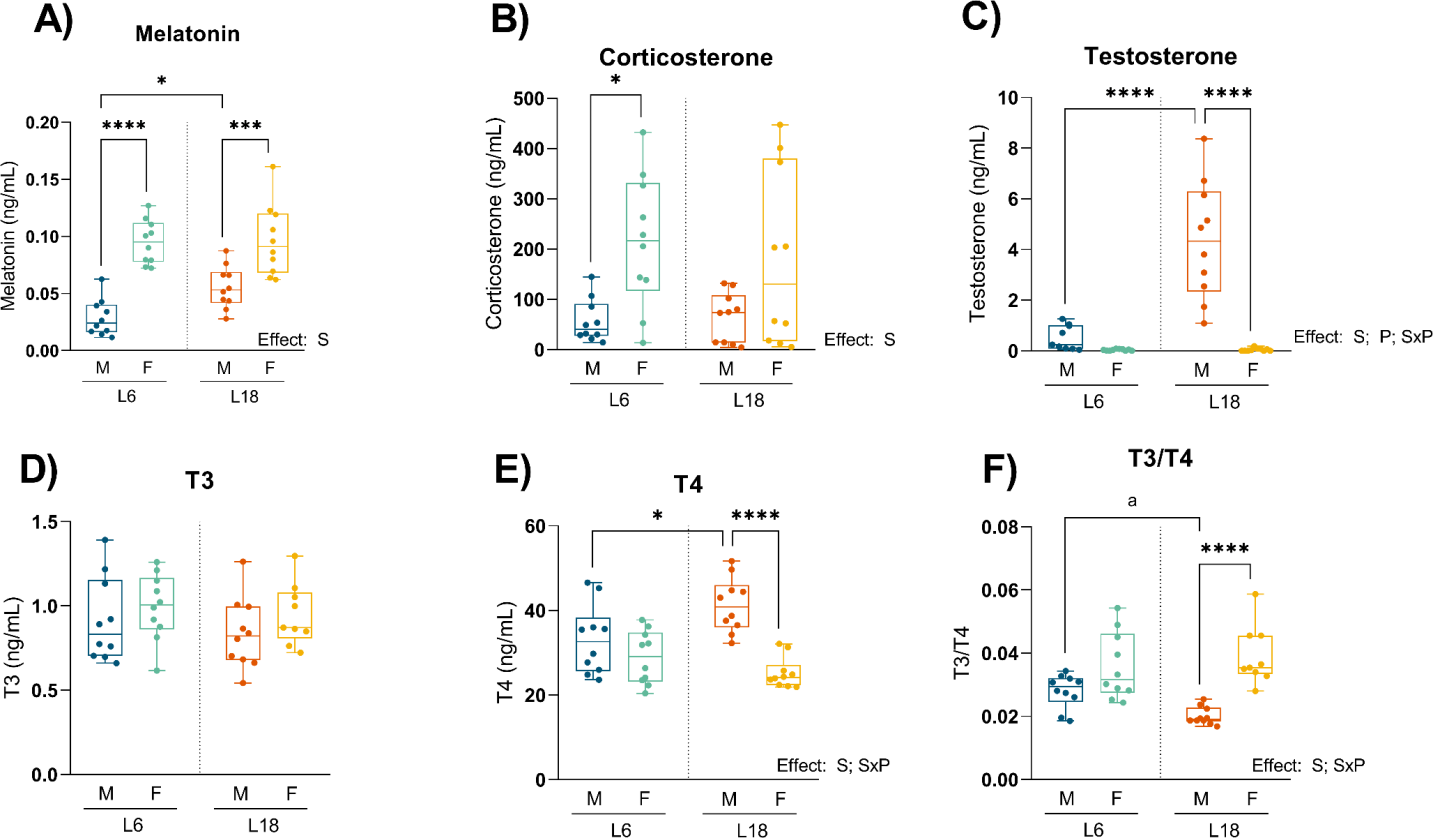
Effect of photoperiod on hormone plasma profile. Melatonin (**A**), corticosterone (**B**), testosterone (**C**), T3 (**D**), T4 (**E**), and T3/T4 ratio (**F**) in males and females F344 rats fed with CAF diet for 11 weeks for the last 8 weeks to short photoperiod of 6/18 h light/darkness (L6) and long photoperiod of 18/6 h light/darkness (L18). Data are expressed as minimum to maximum values, median and interquartile range (*n* = 10). S, sex effect; P, photoperiod effect; SxP, interaction between sex and photoperiod using 2-way ANOVA and Tukey as post-hoc comparisons; * Indicates significant differences by groups (* *p* < 0,05, ** *p* < 0,01, *** *p* < 0,001, **** *p* < 0,0001); a Indicates trend (*p* = 0.1–0.051) using 2-way ANOVA and Tukey as post-hoc test. *M*, males; *F*, females

Melatonin levels were studied as the master hormone modulating seasonal changes of photoperiod. Melatonin levels in plasma were significantly affected by sex and photoperiod. In this sense, in both L6 and L18 photoperiods, melatonin was higher in females than in males, being this difference steeper in short photoperiod. In addition, in males, but not in females, there is a photoperiod effect, with higher levels of melatonin during long photoperiod (Fig. 6A).

In addition, while there are any differences in T3 among studied groups, there was a photoperiod effect in T4 in males, in which T4 levels were higher in L18 than in L6, and a sex effect in long photoperiod, in which T4 levels in females were lower than males. In turn, T3/T4 ratio was significantly higher in females when compared to males in long photoperiod (Fig. 6D-F).

Corticosterone was studied as a response of stress conditions. Levels of corticosterone were slightly higher in females than in males in both photoperiods, but only in L6 significant differences were observed. There was not seen a photoperiod effect, indicating that both males and female rats responded in the same way to the photoperiod changes. In both photoperiods, corticosterone levels in females had more deviation (Fig. 6B).

Testosterone, as the main sexual hormone in males, also influences metabolism changes. Testosterone levels were higher in males than in females in both photoperiods and there was a photoperiod effect in males, where testosterone levels were higher in long than in short photoperiod (Fig. 6C).

### Correlations between hormone profile, final body weight, body composition, and lipid and glycemic parameters

**Fig. 7:**
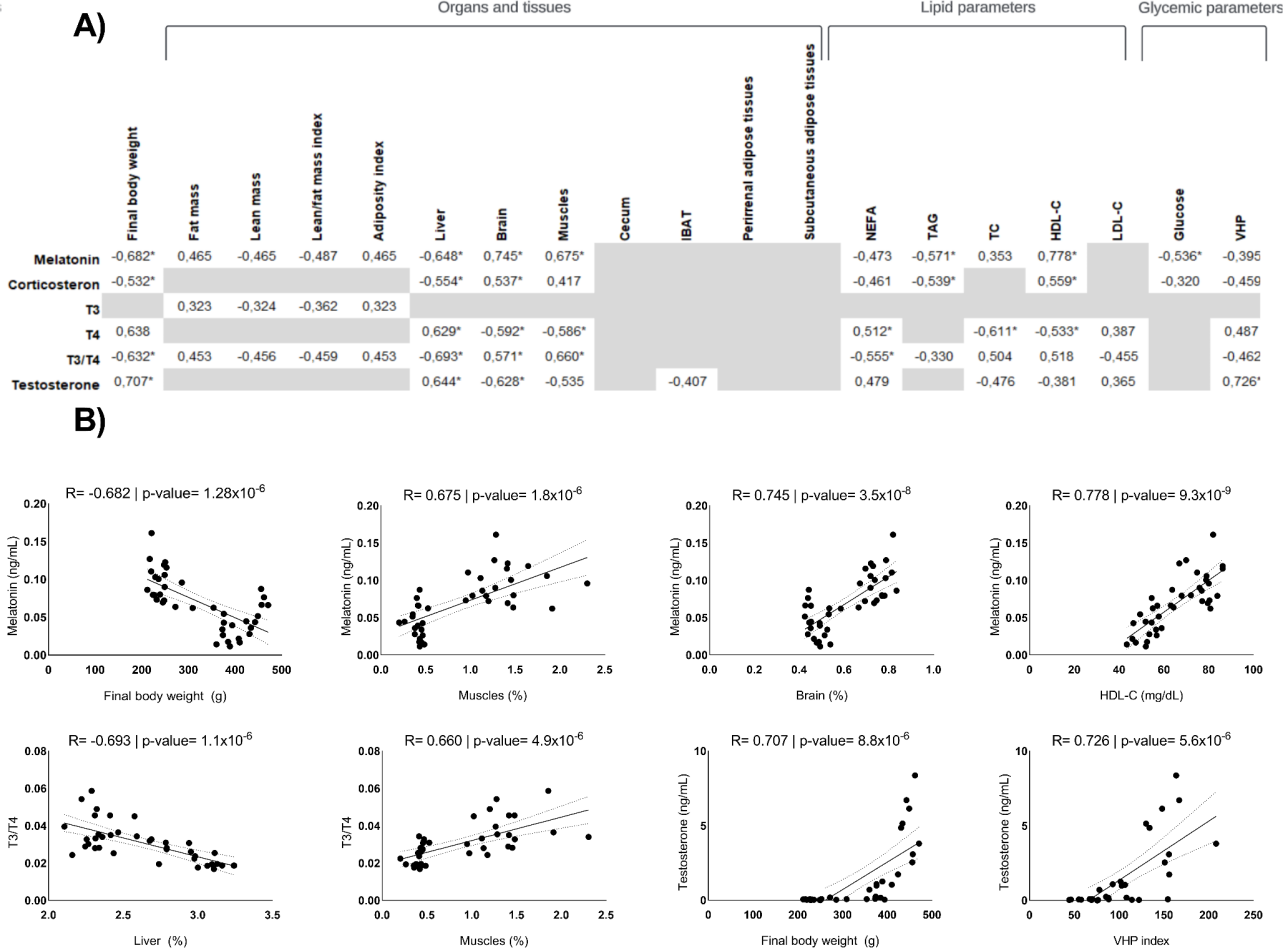
(**A**) Correlation *r* coefficients between circulating hormones and final body weight, organ and tissues’ weight, and lipid and glycemic parameters. Only statistically significant correlations (*p* < 0.05) are shown in the table. * Indicates a significance of *p* < 0.001. (**B**) Linear regression analysis of the strongest observed correlation in parameters affected by sex and photoperiod

Final body weight, body composition, and lipid and glycemic parameters were correlated with circulating hormones. Neither of the hormones studied correlated with the adipose tissues and cecum, while all of them, except T3, correlated with brain, liver or muscles weights (Fig. 7). Melatonin was the hormone with greatest correlation between the studied parameters, modulating them in both positive and inverse ways: while it had a strong inverse correlation with the final body weight, it also affected fat mass and adiposity index in a positive way. Both melatonin and corticosterone had inverse correlation with the glycemic parameters analyzed and with levels of NEFA and TAG, while a positive correlation could be seen in the modulation of HDL-C. Interestingly, neither of both hormones had significant effects in the regulation of LDL-C, which is affected by the action of T4, T3/T4 ratio and testosterone. Testosterone also have a strong positive correlation with final body weight and VHP index.

## Discussion

Humans and other mammals such as F344 rats are seasonal sensitive responding to the different daylight lengths with variations in physiology, metabolism and behaviour. It has been previously reported that the effects of a CAF diet in F344 rats are dependent of the photoperiod [13], and that sex is a determinant factor influencing the physiology and metabolism in obesity [20]. However, to our knowledge, the effect of sex on the responses to different photoperiods conditions has not been studied. Considering the high prevalence of obesity and related pathologies and the influence of photoperiods in physiology, metabolism and behaviour, in this study we aimed to evaluate the impact of sex on daily activity and on the physiological, metabolic and hormonal response to a long and short photoperiod conditions in diet induced photoperiod sensitive F334 rats. For this, rats were fed for 3 weeks with a CAF diet under L12 to allow animals to acclimate and establish weight changes before introducing the photoperiod variable [27]. After this period, male and female CAF fed F344 rats were exposed to a L6 and L18 photoperiods for 8 weeks. The 8-week experimental period under L6 or L18 was selected based on previous studies showing that this duration is sufficient to observe changes in metabolic parameters, body weight, and gene expression related to circadian rhythms and corticosterone levels in F344 rats [28, 29]. In addition, and as previously mentioned, it has been demonstrated that the response of rats when exposed to standard photoperiods with 12 h of light per day is equivalent to long photoperiod exposures, with more than 14 h of light per day, in terms of body weight gain, food intake and gene expression in hypothalamus [7]. F344 rats are commonly used to study photoperiod changes due to their sensitivity to day length variations. Studies with F344 rats can be effectively contrasted with non-photoperiodic strains like Wistar or Sprague-Dawley, which exhibit minimal responses to changes in light [30]. F344 rats exhibit significant physiological and reproductive responses when exposed to different photoperiods. For instance, studies have demonstrated that F344 rats adjust their growth, food intake, and reproductive status in response to varying light exposures [7]. This strain has also shown to exhibit significant alterations in glucose and lipid metabolism in response to different photoperiods [31]. However, one notable concern is the different sensibility to photoperiods of distinct substrains that may involve a variability in responses influencing experimental outcomes [7]. Additionally, F344 rats display genetic insulin resistance from an early age, while maintaining regular levels of glucose, requiring significant higher insulin levels to regulate blood glucose [32].

Different diets are widely used in animal models to induce obesity in rodents, such as diets high in glucose, in fructose, or in lipid content. In this sense, it has been shown that CAF diet could be a robust model to induce human-like MetS in rat models [18]. When feeding F344 rats with a CAF diet, during the summer-simulated photoperiod (L18), both males and females gained more weight than in the winter-simulated photoperiod (L6), although the food intake was lower in both conditions. This normally occurs in mammals, which body weight gain increase during long photoperiods as an adaptative response [33]. The increased weight gain despite reduced food consumption suggests a significant metabolic adaptation to the longer day length possibly involving changes in energy expenditure and storage mechanisms, as it is observed in the increase of EEC in a long photoperiod, in both sexes, compared to L6 conditions [34]. Other study remarked that adiposity tend to decrease in shorter photoperiods when compared to long [14], as evidenced in this study although not statistically significant. In fact, in this study there were no significant differences between photoperiods but between sexes. Specifically, female rats exposed to short photoperiod had increased fat mass and adiposity index but decreased lean mass than males in the same photoperiod conditions. The same study showed that both CAF- and standard (STD)-fed rats in L18 increased lean mass, while only lean/fat mass ratio was lower in CAF-fed rats. In this work, no differences in lean mass between photoperiods can be seen, as referred by Mariné-Casado et al. [13] after 7 week of CAF diet. Our study shows a metabolic adaptation to changes in photoperiods that involve changes in body weight gain and food intake patterns. The higher weight gain in L18 correlates with the lower activity levels that rats presented in this photoperiod compared to L6. In this long photoperiod, animals had fewer hours of darkness and, therefore, fewer hours of the activity phase, resulting in less energy expenditure and more weight gain. The impact of photoperiod on activity is sex dependent, as seen in the differential distribution of activity during L6, with females being more active during the active phase compared to males, while the opposite occurred during the rest phase. This sexual dimorphism in activity patterns suggests that male and female rats have distinct mechanisms for adapting to changes in day length. Activity results suggest that females were not totally adapted to the L6 photoperiod, since their activity decreased significantly after 12 h of darkness. This incomplete adaptation could be due to a mismatch between the imposed photoperiod and the females’ endogenous circadian rhythm, potentially leading to metabolic and behavioral disruptions [3, 35]. Meanwhile, males exhibited a similar activity level during the 18 h of this phase, suggesting a better adaptation to the short photoperiod.

Many studies in L12 photoperiod have confirmed that leptin levels in female rats are higher than in males, due to a greater AI, which results in a higher production rate per unit of mass [36, 37]. However, in the present study, females had lower levels of leptin but higher AI than males in both photoperiods. The increased leptin levels in males correlate with higher daily intake and body weight gain. Males also have a higher insulin resistance in both photoperiods, which results in increased compensatory insulin secretion and thus higher leptin production [38, 39]. Females, on the other hand, demonstrate better insulin sensitivity with lower HOMA-IR index in both photoperiods, but higher under L18 conditions. This may be due to the protective activity of some hormones such as melatonin, which is higher in female rats, or estrogens [40], which play a crucial role in maintaining metabolic homeostasis and protecting against diet-induced metabolic disturbances [41]. Same pattern is shown in males, with higher HOMA-IR index during L18 photoperiod when compared to L6 conditions, although some studies have demonstrated that HOMA-IR index does not vary between photoperiods in males fed with both standard or CAF diet when exposed to L6 or L18 conditions for 9 weeks, highlighting the significant photoperiod-effect shown in this study [42].

Our results confirmed a remarkably effect of sex in lipid parameters. Female rats display a better health status than males regarding lipid parameters, a finding consistent with previous research on sexual dimorphism in metabolic regulation [43, 44]. Females in both photoperiod conditions, exhibited higher levels of HDL-C and lower values of LDL-C than males. This sex-specific difference in lipid profiles aligns with studies showing that estrogens play a protective role in cardiovascular health by modulating lipid metabolism [45, 46]. In addition, compared to male rats, females had higher TC and lower levels of LDL-C at L18 and lower TAG levels at L6, correlating with better lipid metabolism at specific photoperiods. However, there is any difference between photoperiods between rats of the same sex. This lack of a photoperiod-response has been studied by our group in previous studies, where no differences between short, standard, and long photoperiod have been seen regarding lipid parameters in male CAF diet-fed rats [13]. This consistency across photoperiods suggests that lipid metabolism is robust to photoperiod-induced changes, at least in the context of a CAF diet.

Recent studies have shown the relationship between melatonin and lipid plasma profile and fat accumulation, highlighting the role melatonin plays in the improving obesity-related lipid metabolism [47, 48]. Estrogens also have a protective role and are able to affect lipid metabolism both directly and indirectly in females [49, 50]. Directly, they interact with specific estrogen receptors located in various adipose tissues [51]. Indirectly, they modulate the levels of cytokines produced by adipose tissue, which play a fundamental role in various biological processes such as metabolism or inflammation [45]. In this study, there is a clear sex effect in melatonin levels, where females exposed to both photoperiods present higher levels than males, which, along with estrogen levels, may explain the improvement in the lipid profile and insulin resistance when compared to males, thus underscoring the importance of the hormonal profile in the health status of rats [45, 46]. This observation aligns with research on the effects of melatonin on lipid metabolism in rats with diet-induced obesity, which found that chronic continuous melatonin administration reduced weight gain and serum TC levels in standard photoperiod [48]. Melatonin levels significantly modulate various physiological parameters, as evidenced by the observed correlations. This hormone exhibits the highest correlation coefficient concerning its influence on HDL-C levels, while it inversely modulates NEFA and TAG levels, highlighting its protective role in the lipid profile. Additionally, melatonin inversely correlates with final body weight, which was to be expected as females presented higher levels of this hormone and lower body weight at the end of experiment.

Expected levels of melatonin, as a darkness-signaling hormone, in shorter photoperiods should be higher than in long photoperiods as there are more hours of darkness. In this study, there is a photoperiod-effect in males, being higher in L18 conditions, which contradicts the general knowledge. In the experimental design, the cycle of the rats was changed, with the light being switched off at 8:00 a.m., thus at the time of sacrifice, 3 h later, they were in the active phase (darkness). The changes in melatonin levels, and the fact that there is not the expected photoperiod effect, may be due to the timing of sampling, as the samples were not taken at the same time of the dark cycle: at L18 they were taken right in the middle of the active phase, while at L6 they were taken at the beginning of the active phase. This fact can be also related to the lack of differences in T3 hormone among the groups, as it is regulated by changes in melatonin during the dark phase. Nevertheless, changes in T4 can be seen in males between photoperiods, and between sexes in long photoperiods, an increase in males exposed to L18 conditions was notable. In order to obtain a broad overview of the thyroid axis, T3 and T4 were determined as the primary thyroid hormones that directly influence metabolism, energy homeostasis, and reproduction and that are affected by changes in photoperiods [4].

L6 animals exhibited a correlation between NEFA and glucose levels, as both parameters are significantly higher in males than in females. However, L18 females had lower concentrations of NEFA in plasma, but the levels of glucose are similar to males, suggesting an imbalance in glucose metabolism and potentially higher hepatic production [52, 53]. This sex-specific difference in NEFA and glucose levels aligns with research indicating that sex hormones play a crucial role in modulating insulin sensitivity and glucose homeostasis [59, 60].

The association between NEFA and insulin sensitivity has been demonstrated in other studies, with higher NEFA levels often linked to decreased insulin sensitivity [54], as in this study in males. Gómez-Pérez et al. [62] reported no sex-related differences in glucose levels in L12 rats fed with HFD, while insulin levels were higher in males than in females. These results, along with those in this study, suggest that glucose is a sex-dependent parameter in shorter photoperiods, but not in longer ones, such as L12 or L18. Short photoperiods have been shown to enhance glucose utilization in female rats, potentially due to the upregulation of genes involved in insulin signalling and glucose transporter type 4 (GLUT4) mobilization. These molecular adaptations improve glucose uptake in insulin-sensitive tissues like skeletal muscle [31]​. However, insulin levels are sex-dependent in an obesogenic context in all photoperiods.

In long photoperiods, with more hours of light per day, the released of melatonin is lower than in short photoperiods. This hormone inhibits the activity of the axis hypothalamic-pituitary-gonadal (HPG), which oversees liberation of gonadotropin-releasing hormone (GnRH), in charge of testosterone released. It is well reported that, in short photoperiods, as the levels of melatonin are higher, the secretion of testosterone is lower, as referred by Heideman et al. and Tavolaro et al. [7, 55]. This correlates with the results of this study, as the levels of testosterone in males exposed to L18 photoperiod were higher than L6 conditions. The higher presence of this hormone in males under long-day exposure is associated with increased body weight and visceral fat accumulation, as can be seen in the correlation analysis. This is due to testosterone’s role in promoting muscle mass and influencing fat distribution, particularly in the visceral region, which correlates with higher TAG levels and VHP [56]. ​It is well documented that the stress response is sex-based, being females more sensitive to it [57]. Corticosterone is a glucocorticoid hormone that plays a crucial role in the stress response, and it is regulated by the hypothalamic-pituitary-adrenal (HPA) axis. A hormonal imbalance also significantly modulates corticosterone levels, emphasizing the role of the hormonal profile [58]. Otsuka et al. [6] demonstrated that, in normo-weight conditions, corticosterone levels are affected by differences in photoperiods, and the study by Id et al. [59], also showed that corticosterone levels are disrupted by the diet in mice. Corticosterone levels are inversely correlated with body weight gain, and they also influence lipid parameters such as NEFA and TAG in plasma. Interestingly, corticosterone is positively correlated with HDL-C levels, suggesting a potential protective role of this hormone in lipid metabolism, particularly in females. Our results suggest that the sex-effect is more powerful that the effect of photoperiods, as no differences between L6 and L18 have been seen. Female rats exhibit greater variability and higher levels of corticosterone in both short and long photoperiods compared to males. This sex difference is likely due to the increased sensitivity of the HPA axis in females, which is influenced by gonadal hormones and the estrous cycle [60]. In contrast, male rats show lower and more stable corticosterone levels across different photoperiods, suggesting a less reactive HPA axis response to changes in day length [61]. Due to the high variability in female data, care should be taken when interpreting the results, as these fluctuations may reflect underlying complexities not fully accounted for in the current analysis. However, the rest of the data obtained in female rats did not show higher variability than in males, indicating that female rats’ estrous cycle has minimal impact on the validity of the data as reported by Dayton et al. [62].

Overall, the hormonal profile in our study revealed distinct responses between sexes in relation to photoperiod variations. In males, the levels of the studied hormones fluctuated significantly between short and long photoperiods, reflecting an adaptive response to changes in light exposure. These variations in hormone levels were linked to alterations in body weight, fat distribution, and metabolic parameters. However, in females, the response to photoperiod changes was less pronounced, with less significant differences observed in hormone levels between short and long photoperiod conditions. This lack of variation in females may be attributed to the complex interplay of gonadal hormones and the estrous cycle, which could buffer the impact of photoperiod changes on hormone regulation, resulting in a more stable hormonal profile regardless of light exposure [40, 46].

## Conclusion

In conclusion, the present study revealed an interaction between photoperiod and sex, which is associated with differing susceptibilities to diet-induced obesity. This interaction involves sex-dependent differences in daily activity and physiological, metabolic, and hormonal responses to different photoperiod conditions. These findings are of significant physiological relevance since are crucial for understanding how environmental factors, like light exposure, and biological differences, like sex, influences in obesity. This knowledge could ultimately lead to novel and personalized therapeutic strategies or lifestyle recommendations to mitigate obesity, particularly tailored to individual differences related to sex and environmental exposures. However, this study has some limitations such as not analyzing estrogen levels that may lead to miss important data regarding its role in metabolic regulation and the response to diet-induced obesity.

Further research is needed to gain a deeper understanding of how changes in light exposure in obesity impact the molecular mechanisms that regulate circadian rhythms, while also exploring sex differences in these processes. In addition, the analysis of follicle-stimulating hormone (FSH) and luteinizing hormone (LH) may lead to new data on the interaction between photoperiod and sex on reproductive function.

## Data Availability

No datasets were generated or analysed during the current study.

## Abbreviations

ANOVA: Analysis of variance
AI: Atherogenic index
CAF diet: Cafeteria diet
Dio: 2-Deiodinase enzyme type 2
Dio: 3-Deiodinase enzyme type 3
EEC: Energy efficiency coefficient
ELISA: Enzyme-linked immunosorbent assays
F344: Fischer 344
FSH: Follicle-stimulating hormone
GLUT: Glucose transporter type 4
GnRH: Gonadotropin-releasing hormone
HDL: C-High-density lipoprotein cholesterol
HFD: High-fat diet
HOMA: IR-homeostatic model assessment for insulin resistance
HPA: Hypothalamic-pituitary-adrenal axis
HPG: Hypothalamic-pituitary-gonadal axis
HTP: Hypothalamus-pituitary-thyroid axis
IBAT: Interscapular brown adipose tissue
L12: Standard photoperiod, 12 h of light per day
L18: Long photoperiod, 18 h of light per day
L6: Dhort photoperiod, 6 h of light per day
LC: QqQ-Ms/Ms-Liquid chromatography with triple quadrupole tandem mass spectrometry
LDL: C-Low-density lipoprotein cholesterol
LH: Luteinizing hormone
MetS: Metabolic syndrome
NEFA: Non-esterified fatty acids
Q1: First quartile
Q3: Third quartile
SPE: Solid phase extraction
STD diet: Standard diet
T3: Triiodothyronine
T4: Tetraiodothyronine
TAG: Triglycerides
TC: Total cholesterol
VHP: Visceral hypertriglyceridemic phenotype
